# Synthesis, characterization, and pharmacological evaluation of novel azolo- and azinothiazinones containing 2,4-dihydroxyphenyl substituent as anticancer agents

**DOI:** 10.1007/s00706-015-1453-4

**Published:** 2015-04-02

**Authors:** Joanna Matysiak, Małgorzata Juszczak, Monika M. Karpińska, Ewa Langner, Katarzyna Walczak, Marta Lemieszek, Alicja Skrzypek, Wojciech Rzeski, Andrzej Niewiadomy

**Affiliations:** 1 Department of Chemistry, University of Life Sciences in Lublin, Akademicka 15, 20-950 Lublin, Poland; 2 Department of Medical Biology, Institute of Rural Health in Lublin, Jaczewskiego 2, 20-090 Lublin, Poland; 3 Institute of Industrial Organic Chemistry in Warsaw, Annopol 6, 03-236 Warsaw, Poland; 4 Department of Pharmacology, Medical University in Lublin, Chodźki 4a, 20-093 Lublin, Poland; 5 Department of Virology and Immunology, Maria Curie-Skłodowska University in Lublin, Akademicka 19, 20-033 Lublin, Poland

**Keywords:** Azolothiazinones, Resorcinols, Antiproliferative activity, Cytotoxicity, ADMET

## Abstract

**Abstract:**

We reported the synthesis and characterization of a series of azolo- and azino[1,3]thiazinones containing the 2,4-dihydroxyphenyl substituent. The compounds were prepared by a new one-step reaction of aryl-modified sulfinylbis[(2,4-dihydroxyphenyl)methanethione]s and the corresponding aminoazolo(azino)carboxamides. Their chemical structures were confirmed by IR, NMR: ^1^H, ^13^C, HSQC, and EI-MS spectral data. The compounds inhibited proliferation and viability of lung cancer A549, colon cancer HT-29, and glioma C6 cells in a structure- and concentration-dependent manner. The activity of some analogues was below 10 μmol dm^−3^ (IC_50_). Glioma C6 cells were the most sensitive to tested compounds. Generally, the derivatives were not toxic for the skin fibroblast HSF culture. Moreover, some of them exerted a protective effect on the treated normal cells. Evaluation of compound properties in silico showed that they possess significant drug-like characteristics and most of them display a low toxicity.

**Graphical abstract:**

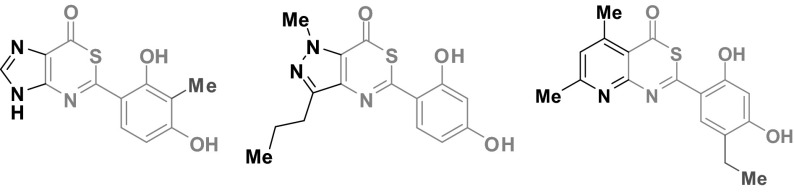

## Introduction

Heterocyclic scaffolds have been a commonly used focal point for the design and development of novel antitumour agents [1–5]. A special group is the derivatives with 2,4-dihydroxyphenyl moiety heat shock protein 90 (Hsp90) inhibitors, for which anticancer activity was very well documented [6]. It includes one five-membered heterocyclic ring of pyrazole [7–11], isoxazole [12], 1,2,3-thiadiazole [13, 14] as well as two fused heterocyclic ring scaffolds of benzisoxazole [15] or 2,3-dihydrobenzoimidazol-2-one [16, 17]. It proved that the resorcinol moiety OH groups are critical to binding with the molecular target [8, 14, 18, 19].

One of the isoxazole resorcinol derivatives, luminespib (NVP-AUY922, VER52296), is a third-generation small-molecule Hsp90 inhibitor with potential antineoplastic activity [20, 21]. It acts via several processes to inhibit tumour growth and metastasis [22, 23]. Luminespib was entered in the I/II phase clinical trials for the patients with advanced solid tumours and multiple myeloma [24–26].

Azolo-1,3-thiazin-4-ones are a group of compounds relatively poorly explored in the area of synthesis and of biological activity. Vicentini et al. described a reaction of trichloromethyl chloroformate with *N*-(1-alkyl/aryl-5-pyrazolyl)thiocarboxamides and *N*-(3-methyl-5-pyrazolyl)thiobenzamide in which pyrazolo[3,4-*d*][1,3] thiazin-4-ones or pyrazolo[1,5-*c*][1,3,5]thiadiazine-4-one were obtained, respectively [27]. Imidazo[4,5-*d*][1,3]thiazine-7(3*H*)-thiones were prepared from 5(4)-substituted amino-4(5)-ethoxycarbonyl-1(3*H*)-imidazoles with the Lawesson reagent [28]. They were designed as acyclovir analogues but exhibited weak biological properties [28, 29]. Other derivatives showed antibacterial [30] or fungicidal activity [31].

The studies carried out by our team of two fused of five- or six-membered rings heterocycles with 2,4-dihydroxyphenyl moiety exhibited an interesting profile of anticancer properties. 4*H*-3,1-benzothiazin-4-ones show high activity against the human bladder cancer HCV29T, non-small cell lung carcinoma A549, breast cancer T47D, and rectal adenocarcinoma SW707 cells [32]. An antiproliferative effect of some analogues is on the level of cisplatin. Structure–activity elucidation exhibited that the presence of a chlorine atom or alkyl substituent (methyl or ethyl) in position 5 of the resorcinol ring has a beneficial effect on the potency of compounds. A similar spectrum of biological activity and analogous effects of resorcinol modification on activity were found for 1,3-thiazolo[5,4-*b*]pyridines [33] and 1*H*-benzimidazoles [34].

In this work to develop new routes for the diversely substituted drug-like heterocyclic scaffolds, we have targeted the 5–6 and 6–6 fused-ring systems of imidazo-, pyrazolo-, and pyridothiazin-4-ones incorporating modified or unmodified 2,4-dihydroxyphenyl substituent. It was assumed that the presence of additional nitrogen atoms compared to 4*H*-3,1-benzothiazin-4-one can enhance interactions with a potential molecular target and intensify biological activity. Antiproliferative effect of the obtained compounds against the cells of some cancer lines and cytotoxicity against normal cells were described. Additionally, ADMET properties in silico of compounds were evaluated.

## Results and discussion

### Chemistry

Using in the reaction with sulfinylbis[(2,4-dihydroxyphenyl)methanethione] (STB) corresponding nucleophiles, heterocyclic carbothioamides possessing an amine group in the neighbouring position to the CONH_2_ group, a set of azolo- and azino**[**1,3]thiazin-4-ones has been obtained. Pyrazolo[3,4-*d*][1,3]thiazin-4(2*H*)-one and pyrazolo[4,3-*d*][1,3]thiazin-7(1*H*)-one scaffolds were obtained from properly substituted 3-amino-1*H*-pyrazole-4-carboxamides **1** and 4-amino-1*H*-pyrazole-5-carboxamides **2**, respectively (Scheme 1). In the reaction with aminoimidazolecarboxamides imidazo-1,3-thiazinones **3** and **4** were formed. The application of 2-aminonicotinamide gave corresponding 4*H*-pyrido[2,3-*d*][1,3]thiazin-4-one (**5**). STB analogues 3Me-STB, 5Me-STB, 5Et-STB, 5Cl-STB, and 3MeO-STB were also applied in the synthesis of the compounds. They possessed substituted benzenediol residue and thus the compounds with modified resorcinol moiety were obtained (Scheme 1).

**Figure:**
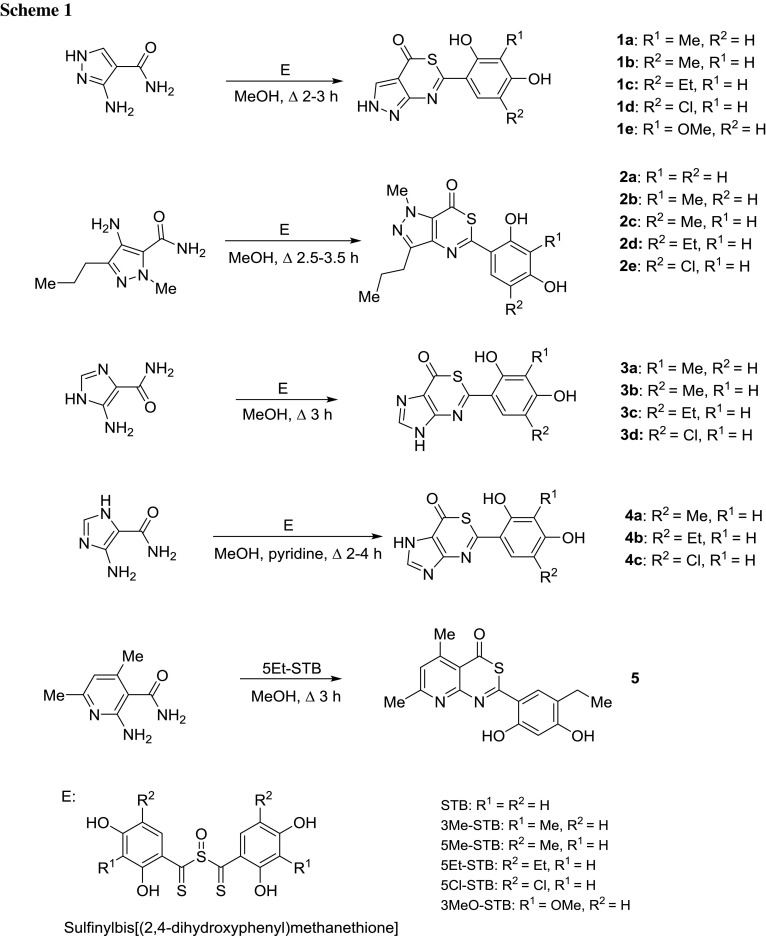

The reagents were applied in equimolar proportions. The reactions were performed in MeOH (sometimes with the addition of pyridine) under reflux (2–4 h) with moderate to good yields (70–88 %). STB and its analogues as the initial reagents were obtained from 2,4-dihydroxybenzenecarbodithioic acid or its analogues and SOCl_2_ in diethyl ether according to the previously presented method [35].

The contents of C, H, and N were within ±0.4 % of the theoretical values. The mass spectra (EI, 70 eV) of all derivatives exhibited the molecular ion peak [M]^+^ of the maximal intensity (*B* = 100 %). The derivatives of group **2** and ethyl analogue **1c** showed the peak at *m*/*z* [M−15]^+^ formed after demethylation of molecular ion.

The IR spectra of compounds exhibited a broad strong band in the range of 3462–3100 cm^−1^ of ν(O–H). A band in the 1692–1649 cm^−1^ range was attributed to C=O group. These spectra confirmed the presence of >C=N– moiety stretching in the region around 1640–1575 cm^−1^. The ^13^C NMR spectra of compounds showed signal at 172–165 ppm attributed to carbon atom of C=O group. Proton of OH group was sometimes invisible (compounds **1b**, **4c**). CH proton of heterocyclic scaffold of all compounds appeared in the range of 8.4–8.0 ppm. NH proton is registered in the wider range of 14.0–12.5 ppm. The cross-peaks of HSQC NMR spectrum of compounds **1b**, **1e**, and **2c** presented in Table 1 further support a formation of new compounds.

**Table 1:**
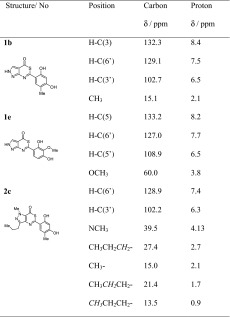
HSQC NMR data of compounds **1b**, **1e**, and **2c**

### Antiproliferative activity

The anticancer effect of compounds was tested in non-small cell lung cancer A549, colon carcinoma HT-29, and glioma C6 cells. The cells were exposed to either culture medium as the control culture or the compounds in concentrations of 10, 25, 50, and 100 μmol dm^−3^. After 96-h treatment MTT assay was performed and IC_50_ (concentration that produced a 50 % viability decrease of cells) values for each cell line were calculated using computerized linear regression analysis of quantal log dose–probit functions [36]. Cisplatin was used as a reference drug.

**Fig. 1:**
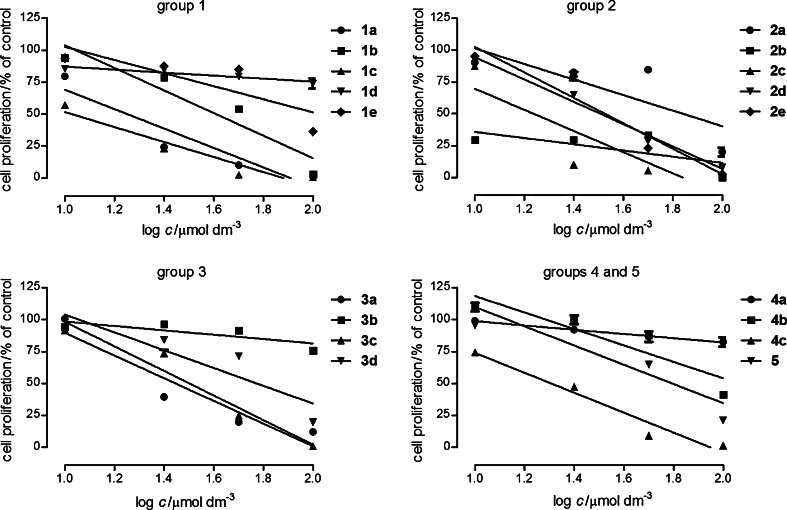
The antiproliferative effect of compounds in rat glioma C6. The cells were grown in the culture medium only (control) and in the presence of tested compounds (10–100 μmol dm^−3^) for 96 h, and the MTT assay was performed. The data represent mean % of control viability ±SEM of six trials and were analyzed by means of linear regression

The tested compounds inhibited proliferation and viability of cells in a structure- and concentration-dependent manner. Glioma C6 cells proved to be the most sensitive to compounds action (Fig. 1). The obtained IC_50_ values are presented in Table 2. The most active were compounds **1a** and **3c** with the IC_50_ values 17.2 and 8.2 μmol dm^−3^, respectively (cisplatin: 0.4 μmol dm^−3^). The evident effect was also detected for compounds **1b**, **1c**, **2c**, **2d**, **3a**, and **4c** (Fig. 1; Table 2).

**Table 2:** Antiproliferative activity of compounds against glioma C6 cells expressed as IC_50_, their molecular descriptors, and parameters of adsorption and distribution processes assumed using ADMET Predictor software

No.	IC_50_/μmol dm^−3^	Log *D*	*M* log *P*	Rule of 5	nRB	PSA/Å^2^	*S*/μg cm^−3^	Peff/cm s^−1^ × 10^−4^	MDCK/cm s^−1^ × 10^−7^	*V*_d_/dm^3^ kg^−1^	BBB	Log BB	PPB/%
**1a**	17.2	2.18	0.95	0	1	99.1	48	2.47	140.73	0.7	Low	−0.29	92.73
**1b**	35.8	2.17	0.95	0	1	99.1	48	2.49	132.27	0.69	Low	−0.35	92.85
**1c**	23.1	2.48	1.22	0	2	99.1	43	2.32	136.17	0.79	Low	−0.27	93.97
**1d**	–^a^	1.75	0.95	0	1	99.1	37	2.88	184.77	0.23	Low	−0.55	98.52
**1e**	100.8	1.57	0.19	0	2	108.0	45	2.94	109.44	0.55	Low	−0.41	92.58
**2a**	48.9	2.69	1.34	0	3	88.2	99	5.18	172.44	0.82	Low	−0.10	96.13
**2b**	–^a^	2.95	1.59	0	3	88.2	82	5.19	196.52	0.91	Low	−0.10	96.64
**2c**	27.1	2.99	1.59	0	3	88.2	80	5.21	186.56	0.90	Low	−0.16	96.76
**2d**	32.2	3.32	1.83	0	4	88.2	70	4.81	193.76	0.97	Low	−0.11	97.32
**2e**	31.8	2.94	1.59	0	3	88.2	74	5.78	249.87	0.38	Low	−0.34	99.04
**3a**	33.1	1.42	0.55	0	1	99.1	51	1.96	110.01	1.07	Low	−0.40	88.69
**3b**	–^a^	1.42	0.55	0	1	99.1	51	1.98	103.09	1.06	Low	−0.46	88.92
**3c**	8.2	1.79	0.82	0	2	99.1	46	1.81	104.61	1.18	Low	−0.37	91.06
**3d**	53.5	0.91	0.55	0	1	99.1	42	2.38	130.67	0.39	Low	−0.65	97.12
**4a**	–^a^	1.39	0.55	0	1	99.1	45	2.41	136.76	0.80	Low	−0.49	91.39
**4b**	89.7	1.76	0.82	0	2	99.1	40	2.26	141.31	0.92	Low	−0.40	92.71
**4c**	18.3	0.76	0.55	0	1	99.1	37	2.78	187.10	0.27	Low	−0.69	97.34
**5**	–^a^	3.35	2.20	0	2	83.3	35	4.94	250.10	1.04	Low	−0.07	97.05

Log *D* octanol–water distribution coefficient, *M* log *P* log *P* according to Moriguchi model, *nRB* number of rotatable bonds, *PSA* Polar Surface Area, *S* water solubility, *Peff* human jejunal effective permeability, *MDCK* apparent permeability for Madin–Darby Canine Kidney (MDCK) cells, *V*_*d*_ volume of distribution, *BBB* qualitative likelihood high/low of crossing the blood–brain barrier, *log BB* logarithm of the brain/blood partition coefficient, *PPB* overall fraction of a drug bound in human blood plasma (in %)

^a^Value was not calculated

**Fig. 2:**
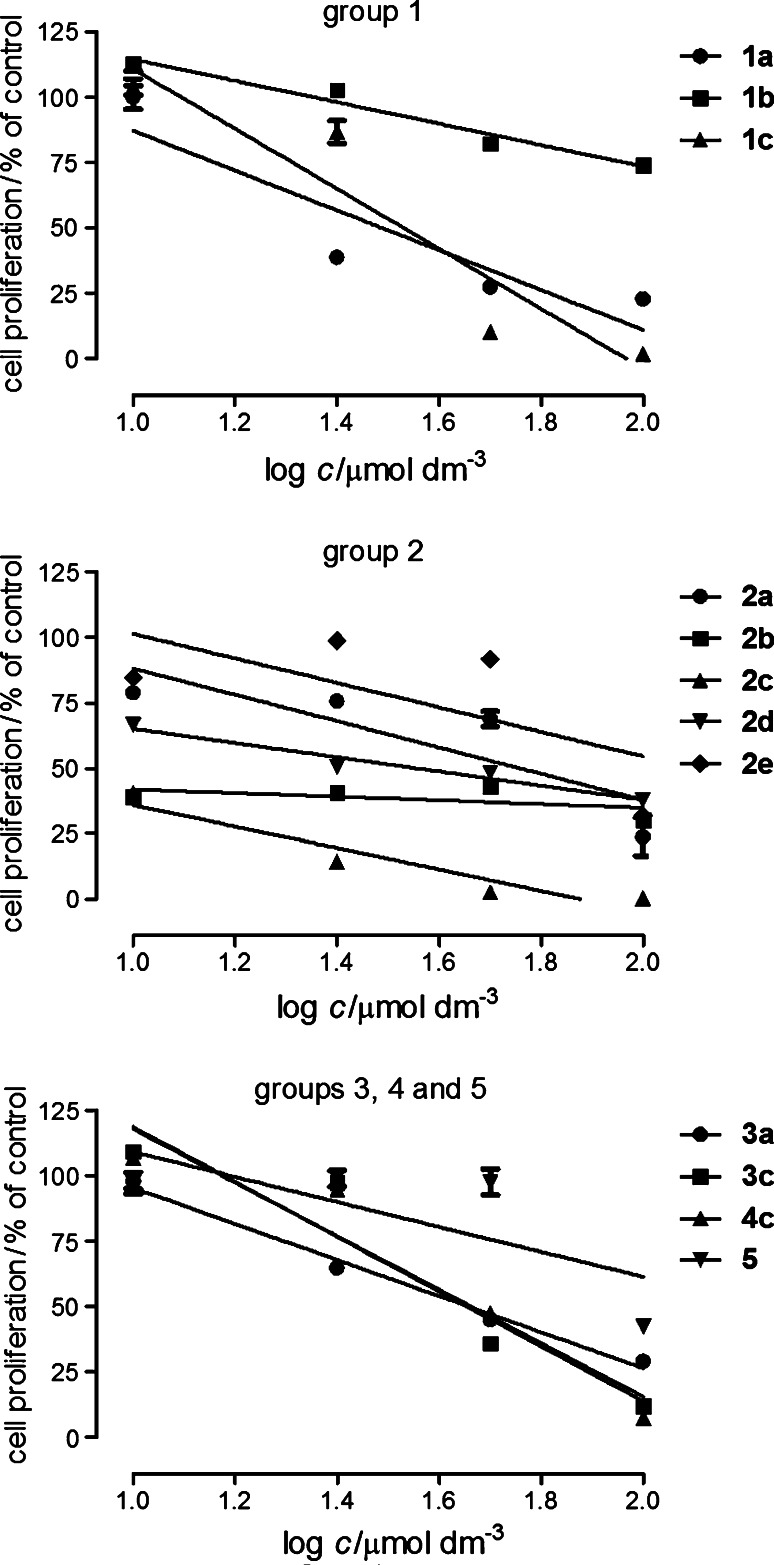
The antiproliferative effect of compounds in human non-small cell lung cancer A549. The cells were grown in the culture medium only (control) and in the presence of tested compounds (10–100 μmol dm^−3^) for 96 h, and the MTT assay was performed. The data represent mean % of control viability ±SEM of six trials and were analyzed by means of linear regression

The compounds under consideration were a little less active against A549 cells (Fig. 2). The most potent were the compounds of group **1**, especially **1a** (IC_50_ = 9.2 μmol dm^−3^). Higher IC_50_ values were calculated for **1c**: 32.2 μmol dm^−3^, **2a**: 56.1 μmol dm^−3^, **2d**: 35.0 μmol dm^−3^, **3a**: 48.8 μmol dm^−3^, **3c**: 49.8 μmol dm^−3^, **4c**: 46.7 μmol dm^−3^ (cisplatin: 4.3 μmol dm^−3^). The other compounds were less active or inactive in the studied concentration range (they are not presented in Fig. 2).

**Fig. 3:**
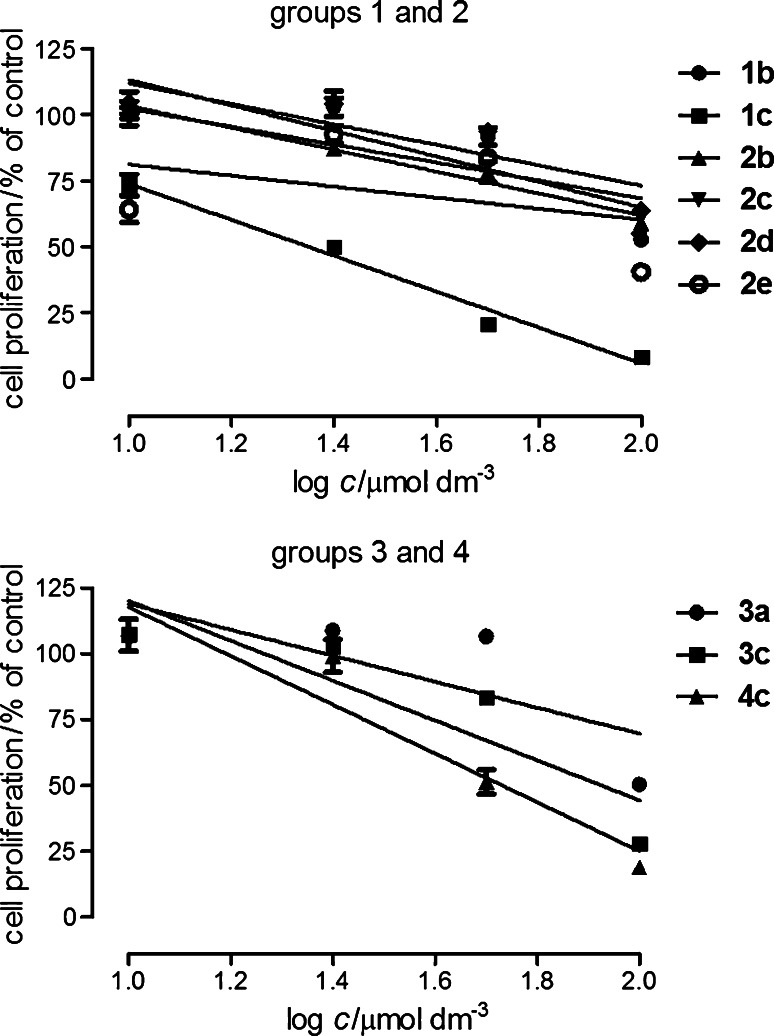
The antiproliferative effect of compounds in human colon carcinoma HT-29. The cells were grown in the culture medium only (control) and in the presence of tested compounds (10–100 μmol dm^−3^) for 96 h, and the MTT assay was performed. The data represent mean % of control viability ±SEM of six trials and were analyzed by means of linear regression

The colon cancer HT-29 cells were the most resistance to the compounds (Fig. 3). The most active were the compounds of group **1**, especially compound **1c** (IC_50_ = 20.9 μmol dm^−3^). Lower activities expressed by IC_50_ were calculated for the following compounds: **1a**: 102.3 μmol dm^−3^, **1b**: 109.3 μmol dm^−3^, **2c**: 157.2 μmol dm^−3^, **3c**: 75.4 μmol dm^−3^, **4c**: 61.4 μmol dm^−3^ (cisplatin: 6.0 μmol dm^−3^). The other compounds inhibited proliferation of HT-29 cells in the studied concentration range to a small extent or did not show antiproliferation effect. Generally, in the tested cancer cell lines the most effective compounds seem to be **1a**, **1c**, **3c**, and **4c**.

**Fig. 4:**
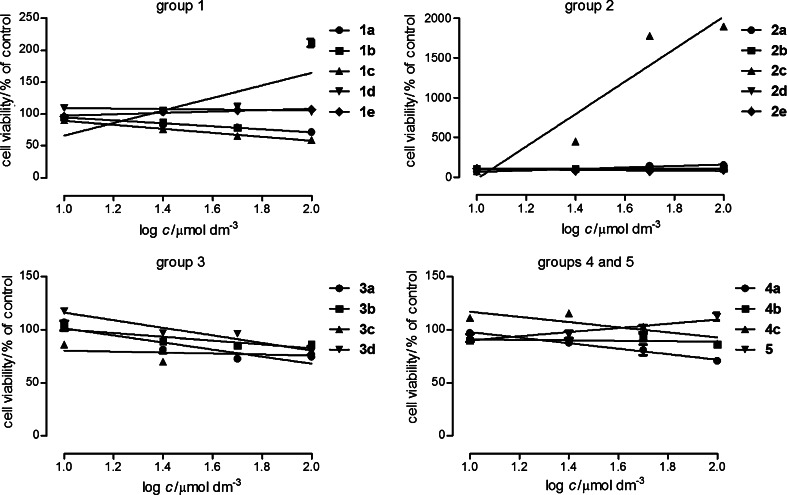
Cytotoxicity of compounds in the primary culture of human skin fibroblasts HSF. The cells were grown in the culture medium only (control) and in the presence of tested compounds (10–100 μmol dm^−3^) for 24 h, and the LDH assay was performed. The data represent mean % of control viability ±SEM of 6 trials and were analyzed by means of linear regression

Additionally, the influence of compounds on normal human skin fibroblast primary culture (HSF) was assessed. HSF cells were exposed to compounds (10–100 μmol dm^−3^) for 24 h and the level of lactic dehydrogenase (LDH) released from the damaged cells was measured (LDH method). The obtained results (Fig. 4) demonstrated that the tested derivatives were relatively low toxic for normal cells. Only compound **2c** in all tested concentrations and **2b** in the concentration 100 μmol dm^−3^ were highly toxic for skin fibroblasts. What is interesting is the fact that compounds **1a**, **1c**, **3a**, **3b**, **3c**, and **4a** induced a decrease of death cells in the culture. This may indicate protective properties of the compounds.

### ADMET properties in silico

The principle goal of the in silico calculation of ADMET properties of compounds is the prediction of their in vivo biokinetics as potential drugs [37, 38]. ADMET Predictor 7.1 was applied to predict ADMET properties of the compounds under consideration [39].

An oral administration is a commonly used route for drugs and a required one for new agents. Absorption of drugs after oral administration is a very complicated process and a number of parameters for its prediction are used. Relatively simple parameters are molecular weight (*M*), hydrophobicity: log *D*, log *P* from different approaches [40], polar surface area (PSA), and a number of hydrogen bonding atoms (HBA, HBD). They are included in the Lipinski’s rule of five [41]. The data presented in Table 2 show that all considered descriptors are in the recommended range (the number of unfulfilled rules = 0). Two other parameters included in the Oprea’s criteria: the number off rotatable bonds (nRB) and polar surface area (PSA) possess also the recommended values (nRB < 10; PSA < 120 Å^2^) [37]. This shows that the compounds under consideration possess strong drug-like properties.

A more sophisticated model of absorption prediction takes into account the values of the human jejunal effective permeability (Peff) and apparent permeability (Papp) for Madin–Darby Canine Kidney (MDCK) cells. The data collected in Table 2 show that for all compounds medium permeability in the range of 103–250 × 10^−7^ cm s^−1^ is assumed [42]. The estimated Peff parameter is larger than 1.8 × 10^−4^ cm s^−1^. The values of both descriptors are the highest for the compounds of group **2** and for analogue **5** which are characterized by the highest lipophilicity and the lowest PSA. Good native water solubility (S) for all compounds is estimated (>10 μg cm^−3^) [43–45].

The PPB parameter was also calculated (Table 2). It describes the overall fraction of a drug bound in human blood plasma (in %). The half of the studied compounds indicate strong binding to proteins of plasma (PPB > 95 %) and for some of them a risk associated with this property (**1d**, **2d**, **2e**, **3d**, **4c**, **5**) is predicted. Two compounds show very weak affinity for them (PPB < 90) (**3a**, **3b**). Generally, a less bounded drug can pass through cell membranes and diffuse more effectively.

Prediction of tissue distribution of a compound is another important task in the drug development. Simple descriptors such as log *P* and *M* are in the optimal range for the studied compounds. The more advanced parameters were also taken into account: volume of distribution (*V*_d_) and the descriptors describing the blood–brain barrier (BBB) penetration [46]. The compounds possess *V*_d_ in the range of 0.23–1.18 dm^3^ kg^−1^, and the highest one was found for compounds **3a**–**3c** and **5**. To predict the BBB penetration, log BB (logarithm of the brain/blood partition coefficient) and BBB filtering (qualitative likelihood high/low of crossing the blood–brain barrier) were calculated. According to the data collected in Table 2 all studied compounds show low brain penetration (log BB < −0.07) and they rather do not across BBB [47].

Anticipating metabolism of compounds we have focused on cytochrome P450 (CYP) enzymes, which are the major enzymes involved in drug metabolism [48]. The following enzymes in human were taken into consideration: CYP 1A2, 2B6, 2C9, 2D6, and 3A4. Table 3 shows that all compounds may be substrates of CYP 1A2 in human and some of 2C9. The data presented in Table 3 show that intrinsic clearance (Clint) due to metabolism mediated by CYP 1A2 in human is significantly lower than via 2C9 enzymes.

**Table 3:** Characteristics of metabolism and toxicity profile of compounds estimated by the ADMET Predictor software

No.	Substrate CYP1A2/%	Clint CYP1A2/µcm^3^ min^−1^ mg^−1^	Substrate CYP2C9/ %	Clint CYP2C9/µcm^3^ min^−1^ mg^−1^	TOX risk	TOX code	TOX MUT risk	TOX MUT code	TOX Rat/mg kg^−1^	ADMET_Risk	ADMET code
**1a**	Yes (63)	4.33	Yes (56)	7.32	1.78	Xm, Hp	1	S3	1372.56	1.78	Xm, Hp
**1b**	Yes (63)	4.20	Yes (56)	3.96	1.92	Xm, Hp	1	S3	1373.27	1.92	Xm, Hp
**1c**	Yes (63)	0.87	Yes (56)	6.27	1.63	Xm, Hp	1	S3	1465.48	1.63	Xm, Hp
**1d**	Yes (57)	3.32	Yes (63)	1.75	1.46	Xm, Hp	1	S3	676.78	2.22	fu, Xm, Hp
**1e**	yes (63)	2.52	Yes (63)	2.07	2.16	Xm, Hp, Mu	2	m1, S3	1105.78	2.16	Xm, Hp, Mu
**2a**	Yes (60)	4.87	No (60)	–	3	Xm, Hp, Mu	2	m1, S3	1342.59	3	Xm, Hp, Mu
**2b**	Yes (60)	7.62	No (64)	–	3	Xm, Hp, Mu	2	m1, S3	1454.44	3	Xm, Hp, Mu
**2c**	Yes (56)	10.30	No (67)	–	3	Xm, Hp, Mu	2	m1, S3	1449.67	3	Xm, Hp, Mu
**2d**	Yes (55)	3.26	No (65)	–	3	Xm, Hp, Mu	2	m1, S3	1474.65	3.16	fu, Xm, Hp, Mu
**2e**	Yes (58)	18.10	Yes (58)	27.90	2	Xm, Hp	1	S3	776.88	4.06	fu, Xm, Hp, 1A, C9
**3a**	Yes (63)	4.99	Yes (75)	36.20	0	–	1	S3	685.69	1	C9
**3b**	Yes (63)	4.99	Yes (56)	23.50	0	–	1	S3	688.09	0.57	C9
**3c**	Yes (63)	0.89	Yes (63)	33.30	0	–	1	m3	716.96	1	C9
**3d**	Yes (58)	6.10	YES (75)	15.00	0	–	1	m3	487.5	0.06	fu
**4a**	Yes (63)	5.92	Yes (63)	18.10	1	Hp	1	S3	998.28	1.21	Hp, C9
**4b**	Yes (63)	1.40	Yes (75)	24.20	1	Hp	1	m3	1056.83	1.62	Hp, C9
**4c**	Yes (57)	5.45	Yes (75)	11.80	1	Hp	1	m3	595.56	1.17	Fu,Hp
**5**	Yes (63)	0.99	Yes (55)	240.00	2	Hp, Mu	2	S1, S3	1315.29	3.03	fu, Hp, Mu, C9

*Xm* TOX BRM Mouse <35 (carcinogenicity in chronic mouse studies), *Hp* hepatotoxicity, *Mu* TOX MUT Risk >1, *fu* % Unbnd <[1,3] (low fraction unbound in plasma), *1A* MET 1A2 km >0.01 μmol dm^−3^ and MET 1A2 CLint >[15,30], *C9* MET 2C9 km >0.01 μmol dm^−3^ and MET 2C9 CLint >[15,30 µcm^3^ min^−1^ mg^−1^], *S1* TOX MUT 97 + 1537 = Positive, *S3* TOX MUT 102 + wp2 = Positive, *m3* TOX MUT m102 + wp2 = Positive AND NOT TOX MUT 102 + wp2 = Positive

Toxicity of compounds, which is a major reason for drug candidate failure, is also estimated. It was presented in two ADMET risk models: TOX MUT Risk and TOX Risk developed by Simulation Plus, Inc. (Table 3) [49]. TOX MUT Risk is a summary of the outputs of the ten different TOX MUT models that independently predict the mutagenicity expected for five strains of *S. typhimurium* with and without microsomal activation. The TOX Risk model consists of seven rules of different toxicities. Table 3 shows that toxicity in Tox Risk model is not predicted for compounds of group **3**. A low toxicity for them is anticipated in Tox Mut Risk model. Predicted toxicity for other compounds is also relatively low and may be connected with carcinogenicity in chronic rat studies (Xm) and hepatotoxicity (Hp) or with mutagenicity in *S. typhimurium.* The calculated acute rat toxicity values (Tox rat) show that compounds are characterized by medium toxicity in the range of 487–1474 mg kg^−1^ predicted for rats after oral administration.

ADMET Risk (global ADMET risk), a computational filter developed also by Simulations Plus Inc [49], was additionally applied. The results collected in Table 3 show that compounds of group **3** with imidazo[4,5-*d*][1,3]thiazin-7(3*H*)-one skeleton, of the lowest lipophilicity in the studied group of compounds, show the best ADMET properties in the group of the studied compounds. Low risk for compounds **4** was also calculated (<1.6). For comparison, ADMET Risk is larger than 6.5 for about 10 % of the drugs focused WDI [49].

## Conclusion

To sum up, we have obtained and characterized a series of azolothiazinones as a new group of heterocyclic compounds possessing the 2,4-dihydroxyphenyl substituent. They were prepared in the one-step novel efficient synthesis procedure. The compounds displayed diverse antiproliferative activities against cancer cell lines. The most sensitive were glioma C6 cells and the most resistant colon carcinoma HT-29. The antiproliferative potency of the most active analogues was below 10 μmol dm^−3^. The majority of the tested compounds were not toxic for normal skin fibroblast culture. Moreover, some of them increased fibroblasts viability. Furthermore, the compounds possess strong drug-like properties and good pharmacokinetics as well as low toxicity is predicted for them in silico. In the light of the presented results, compounds **3c** seem to be most promising. They also provide an opportunity of laying the foundation for development of more promising molecules of anticancer potency.

## Experimental

Melting points were determined using a BÜCHI B-540 (Flawil, Switzerland) melting point apparatus. The elemental analysis (C, H, N) was performed on Perkin-Elmer 2400. The IR spectra were measured with a Perkin-Elmer FT-IR 1725X spectrophotometer (in KBr) or a Varian 670-IR FT-IR spectrometer (ATR) in the range of 600–4000 cm^−1^. NMR spectra were recorded in DMSO-*d*_*6*_ using a Bruker DRX 500 instrument. Chemical shifts (*δ*/ppm) were described in relation to tetramethylsilane (TMS). The MS spectra (EI, 70 eV) were recorded using the apparatus AMD-604.

### *6*-*(2,4*-*Dihydroxy*-*3*-*methylphenyl)pyrazolo[3,4*-*d][1,3]thiazin*-*4(2H)*-*one* (**1a**, C_12_H_9_N_3_O_3_S)

A mixture of 0.177 g 3-amino-1*H*-pyrazole-4-carboxamide (Alfa Aesar, 1.4 mmol) and 0.535 g 3Me-STB (1.4 mmol) in 7 cm^3^ MeOH was heated to reflux for 3 h. The hot mixture was filtered; the formed solid was crystallized from 5 cm^3^ MeOH to give 0.28 g (73 %) yellowish crystals of **1a**. M.p.: 370 °C (dec.); ^1^H NMR (500 MHz, DMSO-*d*_*6*_): *δ* = 12.88 (s, 1H, NH), 12.19 (s, 1H, C(2′)–OH), 10.60 (s, 1H, C(4′)–OH), 8.28 (s, 1H, C(3)–H), 7.53 (d, *J* = 8.8 Hz, 1H, C(6′)–H), 6.56 (d, *J* = 8.8 Hz, 1H, C(5′)–H), 2.04 (s, 3H, CH_3_) ppm; ^13^C NMR (125 MHz, DMSO-*d*_*6*_): *δ* = 164.8, 161.6, 159.0, 155.4, 136.4, 132.3, 126.4, 119.7, 111.2, 110.1, 108.2, 8.0 ppm; IR (ATR): $\bar{\nu }$ = 3213 (OH), 3117 (OH), 1661 (C=O), 1619 (C=N), 1567 (C=C), 1526 (C=C), 1477, 1326, 1252 (C–OH), 1102, 1072, 961, 929, 753 cm^−1^; MS (70 eV): *m*/*z* (%) = 275 (M^+^, 100), 247 (6), 192 (34), 167 (32), 151 (17), 150 (17), 127 (7), 126 (10), 122 (7), 120 (8), 110 (13), 94 (6), 77 (9), 65 (9).

### *6*-*(2,4*-*Dihydroxy*-*5*-*methylphenyl)pyrazolo[3,4*-*d][1,3]thiazin*-*4(2H)*-*one* (**1b**, C_12_H_9_N_3_O_3_S)

A mixture of 0.177 g 3-amino-1*H*-pyrazole-4-carboxamide (Alfa Aesar, 1.4 mmol) and 0.535 g 5Me-STB (1.4 mmol) in 7 cm^3^ MeOH was heated to reflux for 3 h. The reaction mixture was left at room temperature (24 h) and filtered. The formed solid was combined with that obtained after the filtrate concentration. The formed solid was crystallized from 4 cm^3^ MeOH to give 0.33 g (86 %) yellow crystals of **1b**. M.p.: 230 °C (dec.); ^1^H NMR (500 MHz, DMSO-*d*_*6*_): *δ* = 13.12 (s, 1H, NH), 10.59 (s, 1H, C–OH), 8.38 (s, 1H, C(3)–H), 7.52 (s, 1H, C(6′)–H), 6.44 (s, 1H, C(3′)–H), 2.09 (s, 3H, CH_3_) ppm; ^13^C NMR (125 MHz, DMSO-*d*_*6*_): *δ* = 164.7, 162.6, 158.8, 155.4, 136.6, 132.3, 129.1, 122.1, 117.0, 111.1, 102.7, 15.1 ppm; MS (70 eV): *m*/*z* (%) = 276 ([M+1]^+^, 30), 275 (M^+^, 95), 258 (17), 142 (8), 217 (9), 206 (36), 167 (19), 151 (36), 150 (30), 127 (18), 126 (100), 110 (59), 109 (92), 83 (8), 80 (18), 77 (8), 69 (14), 64 (26), 52 (53), 44 (22), 43 (26), 40 (12).

### *6*-*(5*-*Ethyl*-*2,4*-*dihydroxyphenyl)pyrazolo[3,4*-*d][1,3]thiazin*-*4(2H)*-*one* (**1c**, C_13_H_11_N_3_O_3_S)

A mixture of 0.177 g 3-amino-1*H*-pyrazole-4-carboxamide (Alfa Aesar, 1.4 mmol) and 0.575 g 5Et-STB (1.4 mmol) in 7 cm^3^ MeOH was heated to reflux for 2.5 h. The hot mixture was filtered; the formed solid was crystallized from 5 cm^3^ MeOH to give 0.33 g (82 %) dark yellow crystals of **1c**. M.p.: 292–293 °C; ^1^H NMR (500 MHz, DMSO-*d*_*6*_): *δ* = 14.24 (s, 1H, NH), 11.96 (s, 1H, C(2′)–OH), 10.54 (s, 1H, C(4′)–OH), 8.18 (s, 1H, C(3)–H), 7.49 (s, 1H, C(6′)–H), 6.44 (s, 1H, C(3′)–H), 2.53 (m, 2H, *CH*_2_CH_3_), 1.13 (t, 3H, *J* = 7.48 Hz, CH_2_ *CH*_3_) ppm; ^13^C NMR (125 MHz, DMSO-*d*_*6*_): *δ* = 164.7, 161.3, 158.8, 155.4, 136.0, 132.3, 128.0, 123.5, 119.7, 111.1, 102.9, 22.0, 14.1 ppm; IR (KBr): $\bar{\nu }$ = 3471 (OH), 3200 (OH), 3105 (OH), 2965 (CH), 1685 (C=O), 1630 (C=N), 1567 (C=C), 1532 (C=C), 1493, 1458, 1407, 1338, 1267, 1249 (C–OH), 1206, 1185, 1143, 1077, 976, 944, 898, 805, 742,731 cm^−1^; MS (70 eV): *m*/*z* (%) = 289 (M^+^, 100), 274 ([M–CH_3_]^+^, 93), 232 (6), 220 (15), 181 (5), 165 (13), 148 (8), 127 (12), 110 (5), 91 (5), 69 (18), 65 (8), 52 (5), 45 (5), 39 (8).

### *6*-*(5*-*Chloro*-*2,4*-*dihydroxyphenyl)pyrazolo[3,4*-*d][1,3]thiazin*-*4(2H)*-*one* (**1d**, C_11_H_6_ClN_3_O_3_S)

A mixture of 0.177 g 3-amino-1*H*-pyrazole-4-carboxamide (Alfa Aesar, 1.4 mmol) and 0.593 g 5Cl-STB (1.4 mmol) in 7 cm^3^ MeOH was heated to reflux for 2 h. The hot mixture was filtered and the filtrate was concentrated. The formed solid was crystallized from 4 cm^3^ MeOH to give 0.33 g (79 %) yellow crystals of **1d**. M.p.: 359–360 °C; ^1^H NMR (500 MHz, DMSO-*d*_*6*_): *δ* = 11.81 (s, 1H, NH), 11.26 (s, 1H, C(2′)–OH), 11.02 (s, 1H, C(4′)–OH), 8.10 (s, 1H, C(3)–H), 7.78 (s, 1H, C(6′)–H), 6.63 (s, 1H, C(3′)–H) ppm; ^13^C NMR (125 MHz, DMSO-*d*_*6*_): *δ* = 164.2, 161.3, 158.8, 154.4, 137.1, 138.7, 132.3, 122.2, 112.7, 111.9, 104.2 ppm; IR (ATR): $\bar{\nu }$ = 3444 (OH), 3552 (OH), 3068 (OH), 2846 (CH), 1649 (C=O), 1584 (C=N, C=C), 1521 (C=C), 1491, 1445, 1405, 1374, 1317, 1292, 1251 (C–OH), 1203, 1174, 1052, 968, 939, 870, 801, 772, 746, 728 cm^−1^; MS (70 eV): *m*/*z* (%) = 295 (M^+^, 100), 278 (9), 267 (7), 218 (12), 187 (33), 170 (26), 152 (7), 142 (8), 131 (6), 126 (14), 120 (8), 110 (10), 99 (7), 95 (8), 79 (4), 71 (5), 69 (18), 53 (9), 44 (6), 39 (6).

### *6*-*(2,4*-*Dihydroxy*-*3*-*methoxyphenyl)pyrazolo[3,4*-*d][1,3]thiazin*-*4(2H)*-*one* (**1e**, C_12_H_9_N_3_O_4_S)

A mixture of 0.177 g 3-amino-1*H*-pyrazole-4-carboxamide (Alfa Aesar, 1.4 mmol) and 0.580 g 3MeO-STB (1.4 mmol) in 7 cm^3^ MeOH was heated to reflux for 3 h. The hot mixture was filtered, and the formed solid was combined with that obtained after the filtrate concentration. The compound was crystallized from 5 cm^3^ MeOH to give 0.29 g (71 %) light yellow crystals of **1e**. M.p.: 304–305 °C; ^1^H NMR (500 MHz, DMSO-*d*_*6*_): *δ* = 12.59 (s, 1H, NH), 11.07 (s, 1H, C(2′)–OH), 10.09 (s, 1H, C(4′)–OH), 8.17 (s, 1H, C(3)–H), 7.66 (d, *J* = 8.8 Hz, 1H, C(6′)–H), 6.46 (d, *J* = 8.9 Hz, 1H, C(5′)–H), 3.71 (s, 3H, OCH_3_) ppm; ^13^C NMR (125 MHz, DMSO-*d*_*6*_): *δ* = 164.8, 158.4, 154.0, 149.90, 136.1, 133.2, 124.0, 123.0, 117.4, 111.6, 109.6, 60.0 ppm; MS (70 eV): *m*/*z* (%) = 291 (M^+^, 100), 276 (12), 273 (25), 260 (10), 220 (17), 183 (23), 168 (13), 167 (23), 152 (12), 110 (7).

### *5*-*(2,4*-*Dihydroxyphenyl)*-*1*-*methyl*-*3*-*propylpyrazolo[4,3*-*d][1,3]thiazin*-*7(1H)*-*one* (**2a**, C_15_H_15_N_3_O_3_S)

A mixture of 0.255 g 4-amino-1-methyl-3-propyl-1*H*-pyrazole-5-carboxamide (Sigma-Aldrich, 1.4 mmol) and 0.496 g STB (1.4 mmol) in 7 cm^3^ MeOH was heated to reflux for 3 h. The reaction mixture was left at room temperature (24 h) and filtered. The formed solid was combined with that obtained after the filtrate concentration. The compound was crystallized from 5 cm^3^ MeOH to give 0.36 g (87 %) yellow crystals of **2a**. M.p.: 268–269 °C; ^1^H NMR (500 MHz, DMSO-*d*_*6*_): *δ* = 12.53 (s, 1H, C(2′)–OH), 10.37 (s, 1H, C(4′)–OH), 7.54 (d, *J* = 8.81 Hz, 1H, C(6′)–H), 6.41 (dd, *J* = 8.81, 2.33 Hz, 1H, C(5′)–H), 6.36 (d, *J* = 2.33 Hz, 1H, C(3′)–H), 4.13 (s, 3H, CH_3_), 2.77 (t, *J* = 7.39 Hz, 2H, C*H*_*2*_CH_2_CH_3_), 1.73 (sextet, *J* = 7.38 Hz, 2H, CH_2_C*H*_2_CH_3_), 0.94 (t, *J* = 7.38 Hz, 3H, CH_2_CH_2_ *CH*_*3*_) ppm; ^13^C NMR (125 MHz, DMSO-*d*_*6*_): *δ* = 172.4, 163.2, 162.4, 160.0, 146.2, 136.9, 129.9, 122.4, 111.0, 108.6, 103.1, 39.0, 27.1, 21.5, 13.7 ppm; IR (ATR): $\bar{\nu }$ = 3280 (OH), 2940 (CH), 1690 (C=O), 1629 (C=N), 1587 (C=C), 1529 (C=C), 1485, 1442, 1335, 1246 (C–OH), 1217, 1179, 1135, 1106, 1031, 973, 878, 769 cm^−1^; MS (70 eV): *m*/*z* (%) = 317 (M^+^, 100), 302 (21), 289 (75), 280 (13), 256 (9), 242 (15), 214 (20), 173 (21), 153 (10), 119 (7), 69 (6), 42 (8).

### *5*-*(2,4*-*Dihydroxy*-*3*-*methylphenyl)*-*1*-*methyl*-*3*-*propylpyrazolo[4,3*-*d][1,3]thiazin*-*7(1H)*-*one* (**2b**, C_16_H_17_N_3_O_3_S)

A mixture of 0.255 g 4-amino-1-methyl-3-propyl-1*H*-pyrazole-5-carboxamide (Sigma-Aldrich, 1.4 mmol) and 0.535 g 3Me-STB (1.4 mmol) in 7 cm^3^ MeOH was heated to reflux for 2.5 h. The reaction mixture was left at room temperature (24 h) and filtered. The formed solid was crystallized from 5 cm^3^ MeOH to give 0.40 g (86 %) yellowish crystals of **2b**. M.p.: 269–270 °C; ^1^H NMR (500 MHz, DMSO-*d*_*6*_): *δ* = 13.18 (s, 1H, C(2′)–OH), 10.37 (s, 1H, C(4′)–OH), 7.41 (d, *J* = 8.80 Hz, 1H, C(6′)-H), 6.51 (d, *J* = 8.79 Hz, 1H, C(5′)–H), 4.14 (s, 3H, CH_3_), 2.80 (t, *J* = 7.32 Hz, 2H, C*H*_*2*_CH_2_CH_3_), 2.02 (s, 3H, CH_3_), 1.75 (sextet, *J* = 7.33 Hz, 2H, CH_2_C*H*_2_CH_3_), 0.96 (t, *J* = 7.29 Hz, 3H, CH_2_CH_2_ *CH*_*3*_) ppm; ^13^C NMR (125 MHz, DMSO-*d*_*6*_): *δ* = 172.1, 164.2, 160.3, 158.3, 145.9, 136.7, 126.0, 122.6, 111.1, 110.1, 107.8, 39.5, 27.4, 21.4, 13.7, 8.0 ppm; IR (ATR): $\bar{\nu }$ = 3185 (OH), 2965 (CH), 1686 (C=O), 1620 (C=N), 1538 (C=C), 1481, 1433, 1309, 1266 (C–OH), 1236, 1096, 1066, 1032, 901, 798, 778 cm^−1^; MS (70 eV): *m*/*z* (%) = 331 (M^+^, 100), 303 (62), 302 (10), 256 (40), 187 (13), 167 (6), 77 (6).

### *5*-*(2,4*-*Dihydroxy*-*5*-*methylphenyl)*-*1*-*methyl*-*3*-*propylpyrazolo[4,3*-*d][1,3]thiazin*-*7(1H)*-*one* (**2c**, C_16_H_17_N_3_O_3_S)

A mixture of 0.255 g 4-amino-1-methyl-3-propyl-1*H*-pyrazole-5-carboxamide (Sigma-Aldrich, 1.4 mmol) and 0.535 g 5Me-STB (1.4 mmol) in 7 cm^3^ MeOH was heated to reflux for 3 h. The reaction mixture was left at room temperature (24 h) and filtered. The obtained solid was combined with that obtained after the filtrate concentration. The formed solid was crystallized from 5 cm^3^ MeOH to give 0.37 g (79 %) pink crystals of **2c**. M.p.: 260–261 °C; ^1^H NMR (500 MHz, DMSO-*d*_*6*_): *δ* = 12.45 (s, 1H, C(2′)–OH), 10.40 (s, 1H, C(4′)–OH), 7.37 (s, 1H, C(6′)–H), 6.30 (s, 1H, C(3′)–H), 4.13 (s, 3H, CH_3_), 2.74 (t, *J* = 7.44 Hz, 2H, C*H*_*2*_CH_2_CH_3_), 2.07 (s, 3H, CH_3_), 1.73 (sextet, *J* = 7.41 Hz, 2H, CH_2_C*H*_2_CH_3_), 0.95 (t, *J* = 7.40 Hz, 3H, CH_2_CH_2_ *CH*_*3*_) ppm; ^13^C NMR (125 MHz, DMSO-*d*_*6*_): *δ* = 172.2, 163.2, 160.6, 158.2, 146.0, 136.9, 129.0, 122.2, 116.9, 110.2, 102.5, 39.5 (NCH_3_), 27.1, 21.4, 15.1 (CH_3_), 13.8 ppm; IR (ATR): $\bar{\nu }$ = 3166 (OH), 2956 (CH), 1679 (C=O), 1630 (C=N), 1529 (C=C), 1450, 1408, 1382, 1266 (C–OH), 1244, 1137, 1031, 895, 871, 788 cm^−1^; MS (70 eV): *m*/*z* (%) = 331 (M^+^, 100), 316 (13), 303 (51), 302 (8), 256 (11), 228 (15), 187 (13).

### *5*-*(5*-*Ethyl*-*2,4*-*dihydroxyphenyl)*-*1*-*methyl*-*3*-*propylpyrazolo[4,3*-*d][1,3]thiazin*-*7(1H)*-*one* (**2d**, C_17_H_19_N_3_O_3_S)

A mixture of 0.255 g 4-amino-1-methyl-3-propyl-1*H*-pyrazole-5-carboxamide (Sigma-Aldrich, 1.4 mmol) and 0.575 g 5Et-STB (1.4 mmol) in 7 cm^3^ MeOH was heated to reflux for 3.5 h. The reaction mixture was left at room temperature (24 h) and filtered. The formed solid was crystallized from 5 cm^3^ MeOH to give 0.40 g (82 %) yellow crystals of **2d**. M.p.: 269–270 °C; ^1^H NMR (500 MHz, DMSO-*d*_*6*_): *δ* = 12.44 (s, 1H, C(2′)–OH), 10.40 (s, 1H, C(4′)–OH), 7.36 (s, 1H, C(6′)–H), 6.40 (s, 1H, C(3′)–H), 4.13 (s, 3H, CH_3_), 2.78 (t, *J* = 7.40 Hz, 2H, *CH*_*2*_CH_2_CH_3_), 2.50 (t, *J* = 7.47 Hz, 2H, *CH*_*2*_CH_3_), 1.73 (sextet, *J* = 7.4 Hz, 2H, CH_2_ *CH*_*2*_CH_3_), 1.13 (t, *J* = 7.47 Hz, 3H, CH_2_ *CH*_*3*_), 0.95 (t, *J* = 7.34 Hz, 3H, CH_2_CH_2_ *CH*_*3*_) ppm; ^13^C NMR (125 MHz, DMSO-*d*_*6*_): *δ* = 172.2, 163.5, 160.5, 158.2, 146.1, 136.0, 127.8, 123.4, 119.1, 110.8, 102.8, 39.5 (NCH_3_), 27.1, 22.0 (CH_3_ *C*H_2_), 21.5, 14.1 (CH_3_ *C*H_2_), 13.7 ppm; IR (ATR): $\bar{\nu }$ = 3319 (OH), 2968 (CH), 1678 (C=O), 1628 (C=N), 1526 (C=C), 1408, 1339, 1238 (C–OH), 1226, 1126, 1032, 898, 829, 794, 702 cm^−1^; MS (70 eV): *m*/*z* (%) = 345 (M^+^, 100), 330 ([M–CH_3_]^+^, 75), 317 (35), 270 (24), 201 (8), 151 (4), 148 (5), 69 (7).

### *5*-*(5*-*Chloro*-*2,4*-*dihydroxyphenyl)*-*1*-*methyl*-*3*-*propylpyrazolo[4,3*-*d][1,3]thiazin*-*7(1H)*-*one* (**2e**, C_15_H_14_ClN_3_O_3_S)

A mixture of 0.255 g 4-amino-1-methyl-3-propyl-1*H*-pyrazole-5-carboxamide (Sigma-Aldrich, 1.4 mmol) and 0.593 g 5Cl-STB (1.4 mmol) in 7 cm^3^ MeOH was heated to reflux for 3.5 h. The reaction mixture was left at room temperature (24 h) and filtered. The formed solid was crystallized from 5 cm^3^ MeOH to give 0.41 g (84 %) yellowish crystals of **2e**. M.p.: 287–290 °C; ^1^H NMR (500 MHz, DMSO-*d*_*6*_): *δ* = 12.53 (s, 1H, C(2′)–OH), 12.13 (s, 1H, C(4′)–OH), 7.66 (s, 1H, C(6′)–H), 6.60 (s, 1H, C(3′)–H), 4.15 (s, 3H, CH_3_), 2.81 (t, *J* = 7.39 Hz, 2H, *CH*_*2*_CH_2_CH_3_), 1.73 (sextet, *J* = *7*.36 Hz, 2H, CH_2_ *CH*_*2*_CH_3_), 0.95 (t, *J* = 7.40 Hz, 3H, CH_2_CH_2_ *CH*_*3*_) ppm; ^13^C NMR (125 MHz, DMSO-*d*_*6*_): *δ* = 172.8, 161.2, 157.8, 157.3, 146.7, 137.1, 128.7, 122.2, 112.7, 111.9, 104.2, 40.1 (NCH_3_), 27.1, 21.5, 13.7 ppm; IR (ATR): $\bar{\nu }$ = 3364 (OH), 2967 (CH), 2845 (CH), 1692 (C=O), 1628 (C=N), 1587 (C=C), 1536 (C=C), 1496, 1442, 1413, 1391, 1263, 1242, 1211, 1192, 1091, 1050, 1032, 974, 891, 870, 850, 805, 762, 737 cm^−1^; MS (70 eV): *m*/*z* (%) = 351 (M^+^, 100), 323 (76), 290 (7), 276 (9), 248 (13), 207 (17), 187 (7), 153 (5), 126 (4), 82 (6), 69 (9), 42 (10).

### *5*-*(2,4*-*Dihydroxy*-*3*-*methylphenyl)imidazo[4,5*-*d][1,3]thiazin*-*7(3H)*-*one* (**3a**, C_12_H_9_N_3_O_3_S)

A mixture of 0.252 g 5-amino-1*H*-imidazole-4-carboxamide (Acros Organics, 2 mmol) and 0.764 g 3Me-STB (2 mmol) in 10 cm^3^ MeOH was heated to reflux for 3 h. The reaction mixture was left at room temperature (24 h) and filtered. The formed solid was crystallized from 5 cm^3^ MeOH to give 0.37 g (69 %) light brown crystals of **3a**. M.p.: >410 °C; ^1^H NMR (500 MHz, DMSO-*d*_*6*_): *δ* = 10.43 (s, 1H, NH), 10.19 (s, 1H, C(2′)–OH), 10.16 (s, 1H, C(4′)–OH), 8.26 (s, 1H, C(2)–H), 7.37 (s, 1H, C(6′)–H), 6.43 (d, *J* = 8.90 Hz, 1H, C(5′)–H), 2.03 (s, 3H, CH_3_) ppm; ^13^C NMR (125 MHz, DMSO-*d*_*6*_): *δ* = 161.7, 158.6, 154.7, 136.9. 134.9, 133.4, 126.1, 122.6, 111.2, 110.1, 107.4, 8.0 ppm; MS (70 eV): *m*/*z* (%) = 275 (M^+^, 100), 274 (5), 259 (5), 192 (20), 167 (26), 151 (25), 110 (5), 68 (16).

### *5*-*(2,4*-*Dihydroxy*-*5*-*methylphenyl)imidazo[4,5*-*d][1,3]thiazin*-*7(3H)*-*one* (**3b**, C_12_H_9_N_3_O_3_S)

A mixture of 0.252 g 5-amino-1*H*-imidazole-4-carboxamide (Acros Organics, 2 mmol) and 0.764 g 5Me-STB (2 mmol) in 10 cm^3^ MeOH was heated to reflux for 3 h. The reaction mixture was left at room temperature (24 h) and filtered. The formed solid was crystallized from 5 cm^3^ MeOH to give 0.39 g (71 %) yellow crystals of **3b**. M.p.: 361–362 °C; ^1^H NMR (500 MHz, DMSO-*d*_*6*_): *δ* = 13.42 (s, 1H, NH), 12.54 (s, 1H, C(2′)–OH), 10.25 (s, 1H, C(4′)–OH), 8.10 (s, 1H, C(2)–H), 7.40 (s, 1H, C(6′)–H), 6.45 (s, 1H, C(3′)–H), 2.06 (s, 3H, CH_3_) ppm; ^13^C NMR (125 MHz, DMSO-*d*_*6*_): *δ* = 162.0, 158.0, 154.7, 136.9, 134.9, 133.4, 128.8, 122.2, 116.9, 110.4, 102.4, 15.1 ppm; MS (70 eV): *m*/*z* (%) = 275 (M^+^, 100), 259 (13), 248 (6), 215 (9), 206 (16), 167 (62), 151 (16), 110 (6), 69 (5).

### *5*-*(5*-*Ethyl*-*2,4*-*dihydroxyphenyl)imidazo[4,5*-*d][1,3]thiazin*-*7(3H)*-*one* (**3c**, C_13_H_11_N_3_O_3_S)

A mixture of 0.252 g 5-amino-1*H*-imidazole-4-carboxamide (Acros Organics, 2 mmol) and 0.821 g 5Et-STB (2 mmol) in 10 cm^3^ MeOH was heated to reflux for 3 h. The reaction mixture was left at room temperature (24 h) and filtered. The formed solid was crystallized from 5 cm^3^ MeOH to give 0.33 g (88 %) light brown crystals of **3c**. M.p.: 334 °C (dec.); ^1^H NMR (500 MHz, DMSO-*d*_*6*_): *δ* = 13.96 (s, 1H, NH), 12.67 (s, 1H, C(2′)–OH), 10.45 (s, 1H, C(4′)–OH), 8.40 (s, 1H, C(2)–H),7.46 (s, 1H, C(6′)–H), 6.45 (s, 1H, C(3′)–H), 2.53 (m, 2H, *CH*_2_CH_3_), 1.11 (t, 3H, *J* = 7.48 Hz, CH_2_CH_3_) ppm; ^13^C NMR (125 MHz, DMSO-*d*_*6*_): *δ* = 162.5, 160.0, 157.0, 134.7, 133.4, 128.9, 127.3, 123.3, 119.1, 110.4, 102.4, 22.3, 14.0 ppm; MS (70 eV): *m*/*z* (%) = 289 (M^+^, 100), 275 (15), 274 (92), 246 (6), 220 (9), 214 (22), 181 (17), 165 (7), 137 (7), 120 (6), 79 (6), 57 (7).

### *5*-*(5*-*Chloro*-*2,4*-*dihydroxyphenyl)imidazo[4,5*-*d][1,3]thiazin*-*7(3H)*-*one* (**3d**, C_11_H_6_ClN_3_O_3_S)

A mixture of 0.252 g 5-amino-1*H*-imidazole-4-carboxamide (Acros Organics, 2 mmol) and 0.874 g 5Cl-STB (2 mmol) in 10 cm^3^ MeOH was heated to reflux for 3 h. The reaction mixture was left at room temperature (24 h) and filtered. The formed solid was crystallized from 5 cm^3^ MeOH to give 0.47 g (80 %) brown crystals of **3d**. M.p.: >410 °C; ^1^H NMR (500 MHz, DMSO-*d*_*6*_): *δ* = 13.49 (s, broad, 2H, NH, C(2′)–OH), 10.45 (s, 1H, C(4′)–OH), 8.34 (s, 1H, C(2)–H), 7.54 (s, 1H, C(6′)–H), 6.66 (s, 1H, C(3′)–H) ppm; ^13^C NMR (125 MHz, DMSO-*d*_*6*_): *δ* = 165.0, 157.5, 154.7, 134.8, 133.4, 130.3, 128.8, 120.5, 117.4, 111.7, 103.4 ppm; MS (70 eV): *m*/*z* (%) = 295 (M^+^, 100), 261 (14), 260 (15), 235 (13), 187 (19), 165 (7), 171 (14), 153 (7), 69 (7).

### *5*-*(2,4*-*Dihydroxy*-*5*-*methylphenyl)imidazo[4,5*-*d][1,3]thiazin*-*7(1H)*-*one* (**4a**, C_12_H_9_N_3_O_3_S)

A mixture of 0.189 g 4-amino-1*H*-imidazole-5-carboxamide (Fluorochem, 1.5 mmol) and 0.574 g 5Me-STB (1.5 mmol) in 7 cm^3^ MeOH and 0.7 cm^3^ pyridine was heated to reflux for 3 h. The reaction mixture was left at room temperature (24 h) and filtered. The obtained solid was combined with that prepared after the filtrate concentration. The formed solid was crystallized from 5 cm^3^ MeOH to give 0.29 g (70 %) brown crystals of **4a**. M.p.: 362–363 °C; ^1^H NMR (500 MHz, DMSO-*d*_*6*_): *δ* = 13.42 (s, 1H, NH), 11.54 (s, 1H, C(2′)–OH), 10.29 (s, 1H, C(4′)–OH), 8.11 (s, 1H, C(2)–H), 7.50 (s, 1H, C(6′)–H), 6.45 (s, 1H, C(3′)–H), 2.06 (s, 3H, CH_3_) ppm; ^13^C NMR (125 MHz, DMSO-*d*_*6*_): *δ* = 164.5, 161.1, 157.8, 154.0, 136.5, 133.5, 130.0, 125.5, 120.1, 116.5, 115.2, 15.1 ppm; IR (ATR): $\bar{\nu }$ = 3338, 3186 (OH), 1670 (C=O), 1616 (C=N), 1599 (C=C), 1466, 1419, 1347, 1263 (C–OH), 1151, 1112, 1031, 820, 770, 742 cm^−1^; MS (70 eV): *m*/*z* (%) = 275 (M^+^, 100), 206 (23), 167 (22), 151 (31), 126 (5), 109 (5), 69 (7).

### *5*-*(5*-*Ethyl*-*2,4*-*dihydroxyphenyl)imidazo[4,5*-*d][1,3]thiazine*-*7(1H)*-*one* (**4b**, C_13_H_11_N_3_O_3_S)

A mixture of 0.189 g 4-amino-1*H*-imidazole-5-carboxamide (Fluorochem, 1.5 mmol) and 0.616 g 5Et-STB (1.5 mmol) in 7 cm^3^ MeOH was heated to reflux for 2 h. The hot mixture was filtered. The formed solid was crystallized from 4 cm^3^ MeOH to give 0.33 g (77 %) light brown crystals of **4b**. M.p.: 239–240 °C; ^1^H NMR (500 MHz, DMSO-*d*_*6*_): *δ* = 13.46 (s, 1H, NH), 11.53 (s, 1H, C(2′)–OH), 10.22 (s, 1H, C(4′)–OH), 8.13 (s, 1H, C(2)-H), 7.47 (s, 1H, C(6′)–H), 6.45 (s, 1H, C(3′)–H), 2.74 (q, *J* = 7.43 Hz, 2H, *CH*_*2*_CH_3_), 1.10 (t, *J* = 7.40 Hz, 3H, CH_2_ *CH*_3_) ppm; ^13^C NMR (125 MHz, DMSO-*d*_*6*_): *δ* = 164.2, 161.2, 154.0, 136.5, 136.1, 134.4, 127.4, 123.4, 119.1, 110.8, 102.8, 22.1, 14.2 ppm; IR (ATR): $\bar{\nu }$ = 3467 (OH), 3218 (OH), 2968 (CH), 2837 (CH), 1675 (C=O), 1620 (C=N), 1599 (C=C), 1443, 1379, 1237 (C=OH), 1136, 1118, 1032, 830, 794, 767, 716 cm^−1^; MS (70 eV): *m*/*z* (%) = 289 (M^+^, 94), 274 ([M–CH_3_]^+^, 100), 246 (7), 220 (10), 214 (21), 204 (6), 181 (15), 165 (15), 148 (7), 123 (9), 109 (7), 77 (9), 69 (15), 43 (9), 39 (8).

### *5*-*(5*-*Chloro*-*2,4*-*dihydroxyphenyl)imidazo[4,5*-*d][1,3]thiazine*-*7(1H)*-*one* (**4c**, C_11_H_6_ClN_3_O_3_S)

A mixture of 0.189 g 4-amino-1*H*-imidazole-5-carboxamide (Fluorochem, 1.5 mmol) and 0.635 g 5Cl-STB (1.5 mmol) in 7 cm^3^ MeOH and 0.7 cm^3^ pyridine was heated to reflux for 3 h. The reaction mixture was left at room temperature (24 h) and filtered. The formed solid was crystallized from 5 cm^3^ MeOH to give 0.31 g (71 %) brown crystals of **4c**. M.p.: >410 °C (dec.); ^1^H NMR (500 MHz, DMSO-*d*_*6*_): *δ* = 11.06 (s, 1H, OH), 8.35 (s, 1H, C(2)-H), 7.50 (s, 1H, C(6′)–H), 6.64 (s, 1H, C(3′)–H) ppm; ^13^C NMR (125 MHz, DMSO-*d*_*6*_): *δ* = 164.5, 161.1, 157.0, 136.5, 136.5, 134.8, 129.6, 124.5, 112.8, 111.9, 103.3 ppm; IR (ATR): $\bar{\nu }$ = 3343, 3200 (OH), 2842 (CH), 1592 (C=O), 1545 (C=C), 1529 (C=C), 1447, 1418, 1350, 1305, 1250, 1175, 1111, 1031, 810, 708 cm^−1^; MS (70 eV): *m*/*z* (%) = 295 (M^+^, 100), 267 (6), 260 (24), 235 (16), 207 (7), 187 (18), 180 (11), 173 (8), 171 (24), 160 (10), 153 (8), 144 (13), 142 (12), 126 (18), 109 (13), 79 (11), 69 (18), 64 (23), 52 (10), 39 (6).

### *2*-*(5*-*Ethyl*-*2,4*-*dihydroxyphenyl)*-*5,7*-*dimethyl*-*4H*-*pyrido[2,3*-*d][1,3]thiazine*-*4*-*one* (**5**, C_17_H_16_N_2_O_3_S)

A mixture of 0.248 g 2-amino-4,6-dimethylnicotinamide (Sigma-Aldrich, 1.5 mmol) and 0.616 g 5Et-STB (1.5 mmol) in 10 cm^3^ MeOH was heated to reflux for 3 h. The hot mixture was filtered. The filtrate was concentrated and the formed solid was crystallized from 4 cm^3^ MeOH to give 0.34 g (68 %) orange crystals of **5**. M.p.: >410 °C; ^1^H NMR (500 MHz, DMSO-*d*_*6*_): *δ* = 14.27 (s, 1H, C(2′)–OH), 10.29 (s, 1H, C(4′)–OH), 8.06 (s, 1H, C(2)–H), 7.89 (s, 1H, C(6′)–H), 6.45 (s, 1H, C(3′)–H), 2.53 (q, *J* = 7.48 Hz, 2H, *CH*_2_CH_3_), 2.39 (s, 3H, CH_3_), 2.31 (s, 3H, CH_3_), 1.13 (t, *J* = 7.48 Hz, 3H, CH_2_ *CH*_3_) ppm; ^13^C NMR (125 MHz, DMSO-*d*_*6*_): *δ* = 168.8, 153.3, 152.2, 147.8, 136.2, 136.0, 128.1, 128.7, 123.1, 123.2, 119.7, 111.2, 102.2, 22.0, 21.0, 19.0, 14.1 ppm; IR (ATR): $\bar{\nu }$ = 3299, 3131 (OH), 2967 (CH), 1650 (C=O), 1600 (C=N), 1556 (C=C), 1491, 1430, 1390, 1349, 1278, 1249 (C–OH), 1217, 1140, 1032, 900, 874, 732, 680 cm^−1^; MS (70 eV): *m*/*z* (%) = 328 (M^+^, 100), 311 (21), 296 (12), 285 (32), 283 (7), 181 (13), 165 (28), 164 (11), 148 (28), 121 (16), 119 (10), 105 (7), 80 (9), 77 (22), 69 (23), 65 (12), 64 (12), 40 (8), 39 (12).

### Cell cultures

Human non-small cell lung cancer A549 (Cat. No. 86012804), human colon adenocarcinoma HT-29 (Cat. No. 91072201), rat glioma C6 (Cat. No. 92090409) cell lines were obtained from the PHE Culture Collections (Public Health England Culture Collections, Porton Down, Salisbury, UK). Primary culture of normal human skin fibroblasts (HSF) was obtained by the outgrowth technique from skin explants of young persons in our laboratory. The cells were kept in the following culture media purchased from Sigma (Sigma Chemicals, St. Louis, MO, USA): A549—3:1 mixture of DMEM and Nutrient mixture F-12 Ham; C6 and HSF—DMEM, HT-29—1:1 mixture of DMEM and Nutrient mixture F-12 Ham. All media were supplemented with 10 % FBS (Sigma), penicillin (100 U cm^−3^, Sigma) and streptomycin (100 μg cm^−3^, Sigma). The cultures were kept at 37 °C in a humidified atmosphere of 95 % air and 5 % CO_2_.

### Proliferation assay

Cancer cells were plated on 96-well microplates at a density of 1 × 10^4^ (A549), 3 × 10^4^ (HT-29), and 0.5 × 10^4^ (C6) cells cm^−3^. Next day, the culture medium was removed and the cells exposed to serial dilutions of compounds (10, 25, 50, and 100 μmol dm^−3^) in a fresh medium. Cell proliferation was assessed after 96 h using the MTT method in which the yellow tetrazolium salt (MTT) is metabolized by viable cells to purple formazan crystals. The cancer cells were incubated for 3–4 h with MTT solution (5 mg cm^−3^). Formazan crystals were solubilized overnight in the SDS buffer (10 % SDS in 0.01 mol dm^−3^ HCl) and the product quantified spectrophotometrically by measuring absorbance at the 570 nm wavelength using a Elx800 microplate reader (BIO-TEK, Highland Park, Winooski, Vermont, USA).

### Cytotoxicity assay

Skin fibroblasts HSF were plated on 96-well microplates at a density of 1 × 10^5^ cells cm^−3^. The following day, the culture medium was removed and the cells exposed to serial dilutions of compounds (10, 25, 50, and 100 μmol dm^−3^) diluted in a fresh culture medium with a reduced amount of FBS (2 %). Cytotoxicity was detected after 24 h with the use In Vitro Toxicology Assay Kit, Lactic Dehydrogenase based (Sigma). The assay is based on the reduction of NAD by the action of lactic dehydrogenase (LDH) released from damaged cells. The resulting NADH is utilized in the stoichiometric conversion of a tetrazolium dye. The resulting coloured compound is measured spectrophotometrically. The test was carried out according to the kit procedure. The colour product was quantified spectrophotometrically at 450 nm wavelength using an Elx800 microplate reader.

### Statistical analysis

Statistical analyses were performed with the use of GraphPad Prism 5 (GraphPad Software, Inc., La Jolla, CA, USA) and Microsoft Office Excel 2007 computer software.

### In silico ADMET evaluation

In silico ADMET evaluation of compounds was performed by ADMET Predictor version 7.1 [39]. Structures of the compounds were saved in the mol format using Chem Office software. Then, mol files of compounds were uploaded into the ADMET predictor software for further evaluation. All descriptors were estimated at pH 7.4.
